# Supplementary material for: Upregulation of COX4-2 via HIF-1α in Mitochondrial COX4-1 Deficiency
Source: Cells. 2021 Feb 20;10(2):452. doi: 10.3390/cells10020452 (PMC7924049; doi:10.3390/cells10020452)
Supplement: Supplementary file 1 [file cells-10-00452-s001.pdf]

PATHWAY			
GLYCOLYSIS		HYPOXIA	
Gene name	Rank Score	Gene name	Rank score
STC1	26.50	STC1	26.50
ANGPTL4	12.38	ANGPTL4	12.38
TGFBI	8.21	PLIN2	9.39
KDELR3	7.77	SERPINE1	9.00
NT5E	6.87	TGFBI	8.21
GPC4	6.87	ERRFI1	8.04
TALDO1	5.83	KDELR3	7.77
PLOD1	5.74	NDRG1	7.48
DDIT4	5.71	GPC4	6.87
SDC2	5.39	CA12	6.60
AK4	5.27	PLAUR	6.07
TPI1	5.22	S100A4	5.97
PLOD2	4.89	TES	5.83
GCLC	4.82	TGM2	5.73
HK2	4.72	DDIT4	5.71
SDC3	4.61	SLC2A1	5.64
PRPS1	4.50	PDK1	5.61
B4GALT7	4.29	PPP1R3C	5.48
UGP2	4.26	HK1	5.39
B4GALT1	4.16	SDC2	5.39
SLC16A3	4.12	AK4	5.27
B3GALT6	4.07	TPI1	5.22
SOD1	4.02	KLF6	5.10
TXN	3.96	NAGK	4.72
PYGB	3.91	HK2	4.72
CHPF2	3.85	BNIP3L	4.68
VEGFA	3.81	SDC3	4.61
SOX9	3.78	UGP2	4.26
IDH1	3.76	NEDD4L	4.19
EGFR	3.64	B3GALT6	4.07
CHPF	3.58	SRPX	4.00
ERO1A	3.53	SLC25A1	3.99
PGAM1	3.51	VEGFA	3.81
TPBG	3.43	MT2A	3.77
PGK1	3.40	MYH9	3.74
P4HA1	3.37	ALDOC	3.72
PKM	3.16	NDST1	3.69
GYS1	3.04	CSRP2	3.65
FKBP4	2.98	EGFR	3.64
ENO2	2.91	ERO1A	3.53
		GBE1	3.45
		TPBG	3.43
		PGK1	3.40

P4HA1	3.37
AKAP12	3.33
RBPJ	3.31
GCNT2	3.06
GYS1	3.04
GPI	3.04
ENO2	2.91
